# Day clinic and inpatient psychotherapy of depression (DIP-D): qualitative results from a randomized controlled study

**DOI:** 10.1186/s13033-016-0074-6

**Published:** 2016-05-23

**Authors:** Christoph Nikendei, Mirjam Haitz, Julia Huber, Johannes C. Ehrenthal, Wolfgang Herzog, Henning Schauenburg, Ulrike Dinger

**Affiliations:** Department of General Internal Medicine and Psychosomatics, Centre for Psychosocial Medicine, University Hospital Heidelberg, Thibautstrasse 2, 69115 Heidelberg, Germany

**Keywords:** Day clinic psychotherapy, Inpatient psychotherapy, Depression, Qualitative research

## Abstract

**Objective:**

Depressive disorders are among the most common psychiatric disorders. For severely depressed patients, day clinic and inpatient settings represent important treatment options. However, little is known about patients’ perceptions of the different levels of care. This study aimed to obtain an in-depth analysis of depressive patients’ experiences of day clinic and inpatient treatment in a combined clinical setting.

**Methods:**

Following a randomized controlled trial comparing day clinic and inpatient psychotherapy for depression (Dinger et al. in Psychother Psychosom 83:194–195, 2014), a sample of depressive patients (n = 35) was invited to participate in a semi-structured interview during an early follow up 4 weeks after discharge. A qualitative analysis of interview transcripts was performed following the principles of constructivist thematic analysis.

**Results:**

Following analysis, 1355 single codes were identified from which five main categories and 26 themes were derived for both groups. In regard to patient group integration and skill transfer to everyday life, distinct differences could be observed between the day clinic and inpatient group.

**Conclusion:**

While adjustment to therapeutic setting and patient group integration seem to be facilitated by inpatient treatment, the day clinical setting appears to promote treatment integration into patients’ everyday contexts, aiding treatment-related skill transfer to everyday life as well as alleviating discharge from clinic treatment. Further studies on depressive subject groups in day clinic and inpatient treatment should investigate aspects of group cohesion and treatment integration in relation to therapeutic outcome.

## Background

With a life-time prevalence rate of up to 24 % for major depressive episodes [1–3], depressive disorders are among the most common psychiatric disorders. For affected patients, depressive disorders are associated with a significant impairment of mental and physical quality of life [4] as well as with high psychological strain including suicidal behavior [5, 6]. Day clinic and inpatient treatment settings offer important therapeutic options for severely depressed patients. However, little is known about the patients’ perception of the different levels of care [7]. Especially qualitative studies providing an in-depth analysis of patients’ views of day clinic and inpatient psychotherapy are rare, despite offering important insight beyond quantitative questionnaire analysis, [8].

Due to a lack of comparative studies, differential indications and recommendations for day clinic versus inpatient treatment still rely on expert opinion [9]. In its current practice, inpatient psychotherapy is an effective way of treatment for a variety of psychiatric and psychosomatic disorders [10–12]. In German healthcare, indications for inpatient treatment include considerable symptom severity, suicidal risk, an inability to function [9, 13, 14], severe social and domestic conflicts, and a nonresponse to outpatient treatment services.

Day clinic treatment offers several advantages that may explain its growing popularity. The commonly recognized main advantage of the day clinic treatment setting, namely the possibility of providing patients with a combination of intense, multimodal treatment while keeping continuous contact to their home environment and everyday stressors, is at the same time one of its most difficult challenges [13]. The avoidance of inpatient hospitalization not only prevents costs, but introduces an early focus on the reintegration into society. Accordingly, the time division between therapy and the home environment (“half-and-half'') allows patients to maintain social contacts [15], and to continue to care for children and relatives, if necessary [9]. This is in line with treatment models focusing on recovery oriented mental health care [16]. The principle of maintaining close contact to the patients’ home environment and stressors may also facilitate the transfer of therapeutic insight to their normal course of life [14]. Furthermore, the day clinic setting may prevent patient-therapist dependency and inhibit an ‘escape into hospital’ [14], as well as alleviate therapy discharge and re-entry to life outside of the hospital [13]. Accordingly, and in light of the growing economic interest in more cost-efficient treatment options [17], the number of day clinic psychotherapy facilities is increasing [13, 18, 19]. Finally, the multimodal approach allows for very intensive treatment within a relatively short time frame.

So far, most studies examining differences of day clinic and inpatient psychotherapy have investigated mixed patient samples. Lischka et al. [20] assessed patients’ social relationships and their impact on day clinic and inpatient treatment. They were able to show that patients living in non-single households preferred day clinic treatment and that relationships were perceived as more supportive at the end of treatment across settings. In a non-randomized trial, Zeeck and colleagues compared predictors of therapeutic outcome in day clinic and inpatient psychotherapy for a mixed patient group [21]. For day clinic therapy, better outcomes were associated with higher motivation and burden at home, while inpatient outcomes were less favorable if patients’ symptoms were triggered by situations at home. Furthermore, inpatients showed the tendency to foster wishes of being taken care of, thereby increasing dependency at the cost of more autonomous coping with one’s life. In addition, greater patient-therapist dependency was correlated with a less favorable treatment course for inpatients [21].

While most clinical trials focus on symptomatic outcome parameters, qualitative studies offer the possibility of obtaining more detailed impressions of patients’ perceptions of different psychotherapy phases. In a qualitative analysis of patients’ answers to open interview questions, a high acceptance of both day clinic and inpatient treatment, with a slight preference for the day clinic setting, could be shown [22]. A study on day clinic and inpatient psychotherapy using semi-structured interviews revealed that day clinic patients saw the facilitated transfer to everyday life as the most valuable element of their setting [8]. Patients with alcohol addiction saw the day clinic setting’s support of their personal autonomy as particularly beneficial [23]. However, as the stated qualitative studies were no randomized controlled trials (RCTs), inpatient and day clinic samples are not comparable. Furthermore, we are not aware of any previous qualitative studies focusing on depressed patients’ experiences of day clinic and inpatient psychotherapy.

Following a randomized-controlled trail comparing day clinic versus inpatient psychotherapy for depressed patients [24, 25], the aim of the present study was to obtain an in-depth analysis of depressive patients’ experiences with differing levels of care, as patients’ subjective experience and definition of treatment success may differ from the type of changes that are captured by standardized questionnaires [26]. Therefore, all patients recruited in the abovementioned study were invited to participate in a semi-structured interview during an early follow up 4 weeks after discharge.

## Methods

### Participants and procedure

The study was conducted between January 2011 and July 2012. A detailed description of the screening process, the diagnostic assessment and the randomisation procedure can be found elsewhere [24, 25]. Patients were included in the study if they were diagnosed with a current major depressive episode (MDE) or dysthymia, were aged between 18 and 60, and lived within 60 km of the treatment hospital. Exclusion criteria were acute psychotic or bipolar disorder, addiction with current substance abuse, borderline personality disorder, anorexia nervosa, other eating disorders with a binge frequency of more than three times per day and a clinical indication for outpatient psychotherapy. In order to be representative for routine clinical practice, no specifications regarding psychopharmacological medication were made for this study. In total, 44 patients meeting the selection criteria agreed to participate in the randomized trial. After randomization, patients were admitted either to day clinic or inpatient psychotherapy and treated with multimodal psychotherapy for 8 weeks. After waiting-list and during-treatment dropouts, 35 completers (17 day clinic, 18 inpatients, correspondent to 77.3 % completer rate in the day clinic group and 81.8 % completer in the inpatient group) remained for the follow-up assessment 4 weeks following hospital discharge (12 weeks after admission). At 4 week follow-up, all completers participated in a semi-structured interview on their experience of day clinic and inpatient psychotherapy.

### Ethics

The study was conducted following the Code of Ethics of the World Medical Association (Declaration of Helsinki 6th revision, 2008) and registered at the German Clinical Trials Register (DRKS00000550). Written informed consent was obtained from all participants as approved by the local Ethics Committee of the University of Heidelberg (No. S-013/2010).

### Treatment

The study took place in the psychotherapy unit of the Department of General Internal Medicine and Psychosomatics at the University of Heidelberg. Combining day clinic and inpatient treatment, half of the patients on the unit are treated in the day clinic setting while the other half receives inpatient therapy. Within the German health care system, intensive hospital-based psychotherapy treatment (i.e. both inpatient and day-clinic) is indicated after failed previous outpatient treatment attempts and/or in the case of special severity requiring hospitalization. Typically, patients are admitted in the context of an exacerbation posing a threat to their longterm social or economic functioning. All group therapies comprise 50 % inpatient and 50 % day clinic patients. Accordingly, both patient groups are treated by the same therapeutic staff and therapists and receive an equal amount of psychotherapeutic interventions per week. Inpatients stay in one-to-three—bed rooms and are free to leave the unit outside of night hours (11 p.m.–6 a.m.), meal times and therapy sessions. Over the treatment period of 8 weeks, inpatients usually spend 6 weekends at home in order to remain in contact with their home environment. Day clinic patients attend therapy on 5 weekdays from 8 a.m. to 4 p.m. They share one meal (lunch) with their fellow inpatients during the day. In addition to the common rooms open to all patients, they are able to retreat to a quiet room between therapy sessions. Outside of day clinic hours, they may call the therapy unit in times of need or emergency at all times.

Following a primary psychodynamic orientation, treatments are carried out in a multimodal psychotherapy setting with integrated cognitive-behavioral, and systemic treatment components. Patients in this study received individual psychotherapy (1/week), psychodynamic-interactional group psychotherapy (2/week), art therapy (2/week), music therapy (1/week), body-oriented therapy (1/week), social competence training (1/week), and systemic sculpture group psychotherapy (1/week). In addition, couple or family sessions were optional. Scheduled therapy sessions were complemented by weekly psychotherapeutic ward rounds, regular contacts with assigned nurses, daily morning and evening groups led by the therapeutic staff, and individual social counselling.

### Development of leading questions

In line with the COREQ checklist [27], the development of the study’s interview questions and hypotheses was performed on the basis of an in-depth literature review as well as discussion among a team of experts (N = 5; 2 female, 3 male, all of whom experienced in psychotherapy training and research). The interview manual was constructed in a semi-standardised manner [28–31] containing the main open questions followed by encouraging and clarifying questions if required. In accordance with Helfferich [28], the leading questions referred to day clinic- and in-patients’ perceived advantages and disadvantages of their respective setting, patient group integration, treatment success, effects on partnerships, family, social environment, and their occupation as well as achieved skill transfer to everyday life. Following the semi-standardised interview manual, individual face-to-face interviews were audio-taped and conducted by a trained, female interviewer supervised by an experienced tutor and colleagues who had conducted similar studies.

### Qualitative content analysis and statistical analysis

For qualitative analysis, we implemented a constructivist thematic analysis approach. The constructivist approach implies that existing relevant literature, e.g. comparative studies on inpatient and day clinic treatment [21], influences research question development and that the resulting sensitisation primes ensuing data analysis. Thematic analysis (TA) is a pragmatic approach to qualitative analysis focusing on the search for identifiable themes across a dataset [32]. Although it draws on some techniques of grounded theory [33, 34], TA follows a six-phase analysis process allowing more flexibility and alleviating adaption to specific study affordances. After verbatim transcription, open line by line coding of all 35 interviews was conducted to identify recurring topics. Specific sentences (or combinations of sentences) were identified as a code representing the most elemental unit of meaning [35]. The assignment of codes to specific themes was conducted by two independent analysts (CN and JH) using the software MaxQDA (2010 version, VERBI GmbH, Berlin), discussed to reach consensus and adjusted if necessary. Themes were compared and adapted, until overarching relevant themes for both groups (day clinic- and in-patients) could be defined. In a final step, themes were summarized into five relevant categories. In order to identify commonalities and differences between the two participant groups, all codes were analysed for each theme comparing meaning and frequency, and consolidated through a profound expert team discussion. In a final step, topics that were more pronounced among day clinic- vs. in-patients were identified. Descriptive quantitative data were managed with the software package SPSS (IBM SPSS Statistics 20) and presented as mean ± standard deviation (SD) and median with interquartile range as applicable.

## Results

### Participant characteristics

Thirty-five participants consented to participate in the study on a voluntary basis (50 % female; aged: 35.1 ± SD 11.6 years; range 18–55). The main diagnosis for thirty-four patients was a MDE and one patient was primarily diagnosed with dysthymia. The comorbid axis-1 diagnoses, in accordance with the structured clinical interview for diagnostic and statistical manual for mental disorders (DSM-IV; SCID), included anxiety disorders (45.5 %), somatoform disorders (13.6 %), dysthymia (11.4 %), eating disorders (11.4 %) and obsessive–compulsive disorders (6.8 %). In addition, 33.3 % of included patients suffered from a personality disorder. Further details can be found elsewhere [24, 25].

### Main categories and themes

The qualitative analysis of the interviews identified 1355 single codes, from which five main categories and 26 themes were derived for both groups. Table 1 provides an overview of the main categories and themes as defined below for the day clinic and the inpatient group. The number of codes per category and theme is shown in parentheses. Illustrative quotations for main categories and themes are listed in Tables 2 and 3 for each group.

**Table 1:** Summary of main categories and themes after qualitative analysis of patient experience

1. Therapeutic aspects (266)	
*Day clinic patients [D]*	*Inpatients [I]*
* D.1.1. Start of therapy (29)*	*I.1.1. Start of therapy (20)*
* D.1.2. Experience of therapy (61)*	*I.1.2. Experience of therapy (98)*
* D.1.3. Involvement of current domestic conflicts in therapy/Therapy * *focus on social and domestic reality (21)*	*I.1.3. The clinic* —*a safe haven (37)*
2. Patient group experience (322)	
*Day clinic patients [D]*	*Inpatients [I]*
* D.2.1. Learning through interaction (19)*	*I.2.1. Learning through interaction (19)*
* D.2.2. Sharing experiences (29)*	*I.2.2. Sharing experiences (34)*
* D.2.3. Group cohesion and sense of belonging (78)*	*I.2.3. Group coherence and sense of belonging (93)*
* D.2.4. Stress experience (26)*	*I.2.4. Stress experience (24)*
3. Social contacts outside of therapy (120)	
*Day clinic patients [D]*	*Inpatients [I]*
* D.3.1.* *Support and improvement of social contacts/Positive * *interactions with family and friends (31)*	*I.3.1.* *Support and improvement of social contacts/Positive interactions with family and friends (42)*
* D.3.2.* *Social withdrawal (18)*	*I.3.2.* *Social withdrawal (29)*
4. Treatment discharge and going back to everyday life (503)	
*Day clinic patients [D]*	*Inpatients [I]*
* D.4.1. Leaving the unit in the evening (9)*	*I.4.1. Visiting home on the weekends (9)*
* D.4.2. Discharge from treatment (29)*	*I.4.2. Discharge from treatment (27)*
* D.4.3. Going back to everyday life (208)*	*I.4.3*. *Going back to everyday life (221)*
5. Experience of the daily commute and evenings at home (144)	
*Day clinic patients [D]*	*Inpatients [I]*
* D.5.1. Experience of the daily commute (40)*	–
* D.5.2. Daily contact with the home environment(40)*	–

**Table 2:** Qualitative analysis of patient statements: citations related to categories 1 to 4

1. Therapeutic aspects (266)
D.1.1. Start of therapy (29) *“At first it was quite difficult. The first two weeks… yes, the whole “arrival*-*thing,” I just did not know what to do with myself and* ***I wasn’t confident enough yet to approach people*** *.” (1.2)* *“At first I was very insecure…* ***just the insecurity of being in a group*** *, especially in the mornings and evenings during these mood scale exercises, which totally confused me at first.” (1.18)* *“Let’s put it this way: I’m* ***not a group person*** *. So the individual therapy sessions were really important and meaningful for me. In the group session, I just had the problem that my fellow patients didn’t know why I’m here. Accordingly, I* ***couldn’t open up in*** *the way I might have needed to. “ (1.13)*
I.1.1. Start of therapy (20) *“Actually, you spend most of the time in therapy with them and so it’s always* ***a bit easier to open up to them,*** *because of that. I found that in general, in any case… so for some day*-*patients, I often thought that they should be inpatients really… I’ve definitely noticed that the people who were inpatients* ***got along far better among themselves*** *compared to the day patients. You really noticed this. You are* ***much more open*** *with each other and you can talk about some things more easily.” (2.2)* *“During the therapy, it was very strange to start with. In the beginning,* ***you had a week of time to yourself*** *. The intention was, probably, that you get to know the other patients, especially fellow patients, and to have time to yourself. It was* ***very depressing*** *. Because I was in* ***a strange environment, had nothing to do and was supposed to just potter around with myself*** *. That was very weird because usually, in everyday life, I’m always here and there and I was always on the move together with someone else. And here: this calmness.” (2.9)* *“So for the first few weeks* ***it was hard for me to become part of the group*** *.” (2.17)*
D.1.2. Experience of therapy (61) *“I found it* ***incredibly helpful to talk*** *about it and it was a relief, when I realized, it all become a little clearer. I was able to explain things to myself or I could change things.” (1.8)* *“Yes, I had* ***a very close relationship*** *to one of my therapists. I felt very well understood and well taken care of there.” (1.1)* *“Already after the first week, I noticed that I* ***felt comfortable*** *and that it was a place, somehow, where I could feel* ***safe*** *.” (1.18)* *“The routine was* ***good for me*** *and the* ***task of working on myself*** *. At the time, I already noticed that it helps me.” (1.8)* *“Really, everything was exhausting, I think. This was the first time that I ever had to really talk, because, usually, I* ***never talk about problems*** *. And there, you just had to talk, you had to join in, you had to accept this and that. I found that all just very tiring,” (1.17)*
I.1.2. Experience of therapy (98) *“…We’re all going through the same stuff, really. Everyone has their own problems, that’s for sure, but you can simply learn a lot from the others, also from the older ones. And many* ***opened my eyes*** *. So, I guess, the other patients were the best therapy! Definitely.” (2.9)* *“It was all these conversations. Although they upset me,* ***but afterwards it always felt better*** *. I had a much clearer view of things.” (2.16)* *“… I was glad that I was an inpatient. I wouldn’t have been able to guarantee that I would’ve come every day. And so I just knew that breakfast was at seven*-*thirty. It put* ***a little pressure on me*** *. This was beneficial for me.” (2.5)* *“So I thought it was really hard work. After some therapies, I felt really knackered.” (2.12)*
D.1.3. Involvement of current conflicts at home in therapy (21) *“… and if I had difficulties or problems or other things, that I was able to work with them and discuss them* ***right on the very next day.*** *That was very helpful, really very helpful.” (1.6)* *“And it is this being able to use things, that you could* ***use things, you’d learned right away*** *. And not learn stuff for eight weeks and then blast people around you with change.” (1.7)* *“It was also* ***important to see for myself: “Look, you can really do this*** *. I am able to go to work or therapy in the morning and evening and come home again.” (1.5)*
I.1.3. The clinic -a safe haven (37) *“I was also glad that I was away from home. That I was simply out of the whole surroundings and was in a protected environment, where* ***it really was about me for once*** *. Because I have not taken care of myself for 40* *years. But now I had eight weeks of time to realize that I also exist. And* ***I’m not under this pressure to come back home in the evening to feed my family, to do the ironing and washing*** etc*. And especially with my boys at home, it was also important that they used the time to learn to be more independent.” (2.15)* *“I found being inpatient was actually much better than being the other, because you are* ***completely away from home*** *. That is, you are actually* ***worry*** **-** ***free*** *. You have nothing to worry about. Your* ***head is free to focus on yourself*** *. That was something that really helped me an awful lot.” (2.7)* *“And that is, what I think is valuable in inpatient treatment. That you are able to get that* ***necessary distance from home*** *.” (2.6)* *“So, I just think that* ***the reference to normal, everyday life was a bit too small*** *.” (2.4)*

2. Experience of the patient group (322)
D.2.1. Learning through interactions (19) *“And then I simply noticed that here you can really say what you think. That you won’t be judged or demonized for it. I had the* ***feeling of being accepted just as I am*** *. With all the “ifs and buts” and that simply felt incredibly good.” (1.18)* *“It was* ***positive feedback*** *, and that of course brought about* ***a sense of self*** **-** ***worth again*** *… For example, that people said that it was pleasant talking to me. I guess, I just have, for example, a rather bizarre sense of humor and made people laugh from time to time. They simply thought that was great. My* ***self*** **-** ***esteem has gone up*** *.” (1.14)* *“I came in here, and I just started* ***trying everything out*** *with my fears that I otherwise wouldn’t have dared to do. I felt very comfortable and held by my group.” (1.7)*
I.2.1. Learning through interaction (19) *“I then realized that everybody didn’t want to harm me all the time and I started* ***regaining confidence*** *. It definitely helped that you had to deal with the other patients.” (2.2)* *“The feedback that I received. How* ***I come across to people*** *. Because it was always so horrible for me feeling rejected so often.” (2.7)* *“Before therapy, I often felt out of place. I* ***couldn’t carry a conversation anymore*** *and was already thinking, “Oh God, I’m a hermit. I can’t even talk to people anymore.” That made me socially retreat more and more. And here I realized:* ***People like being with you!*** *They like having a chat with you; they are interested in what you have to say. That was a really nice feeling.* ***Because I always thought no one likes hanging out with me*** *. I have nothing to offer, nothing interesting to tell. There’s nothing there, what I could give. And here, I just started seeing it again: there is something there! It was simply hidden or whatever you’d call it.” (2.12)*
D.2.2. Sharing experiences (29) *“There were quite a few discussions, which helped me a lot. Just seeing,* ***there are other perfectly normal people, like me, who are dealing with the same problems.*** *Somehow, this has helped me the most.” (1.2)* *“Here you are able to talk and that really helped me, because they* ***understand you here*** *. Because everyone is dealing with pretty much the same somehow. Here you can* ***talk about stuff you can’t talk about outside or at work*** *.” (1.4)* *“Patients lift each other up a bit, and* ***you can support each other*** *and always find good conversations.” (1.3)*
I.2.2. Sharing experiences (34) *“Outside, you always have to explain so much and here, a few words were enough and you’re understood. That was something special. Nowhere else is like that.* ***A whole load of people, who understand you without you having to say a lot of words.*** *” (2.12)* *“When we sat together in the evening… and just talked about experiences…* ***you could really benefit from the others’ experiences*** *.” (2.6)* *“The ton of time you spend with the other patients after the end of the day’s work.* ***So, really the exchange actually takes place then, when the day patients are gone*** *. That’s when it really begins.” (2.15)* *“It was nice* ***because there was always somebody there, if one was not feeling too good*** *, with whom you could always talk. You did not have to plan anything; that was just great. If you felt the need to, you simply went and joined people somewhere.” (2.12)*
D.2.3. Group cohesion and sense of belonging (78) *“This* ***sense of community*** *, it was there in a way. As I knew it in the past, like it always used to feel.” (1.8)* *“I mean, I was made* ***to feel very welcome*** *and also welcomed other people after a certain time.” (1.4)* *“It was simply a kind of an* ***outsider feeling*** *. The others are there to stay and spend the evening together and get to know each other better and I have to* ***go back home at 4 o’clock*** *. I’m missing out on something. They are having a good time and I’m home alone… the feeling* ***of being a second*** **-** ***class patient and only a guest*** *. Also* ***not really being part of*** ***the community*** *of inpatients….” (1.11)* *“It just really dawned on me. Really, just the thought: “This isn’t helping! It can’t help like this” And:”* ***I don’t belong to the group*** *” that popped up again and again.” (1.11).* *“It was just this* ***feeling of being a stepchild*** *when you’re only a day clinic patient.” (1.11)*
I.2.3. Group coherence and sense of belonging (93) *“It created a great sense of community and gave me a lot of information and input. For me, that was* ***really the best part.*** *” (2.15)* *“As I said, the* ***contact with the other patients was much, much more intense*** *. I wouldn’t want to miss that, really. This sense of togetherness, this feeling of solidarity, I would really have missed it. I’d have felt more like a patient, and this way it felt more* ***like a family*** *.” (2.16)* *“So, for me it was an* ***essential part of therapy*** *(…) if I imagine, I’d have had to go home at 4 o’clock, a large part of the actual therapy would have been missing. Exchanging experiences is incredibly valuable. To be able to interact with others, but not to have to. Or just joining the others while they were chatting, was immensely valuable. The day clinic would not have been an option for me.” (2.13)* *“Then I had the exact same problem which I normally have, I* ***felt excluded*** *from the group of other patients, I felt* ***isolated,*** *I* ***couldn’t get a connection*** *, and it was then that I noticed that this was a general problem and not only to do with the work.” (2.1)*
D.2.4. Stress experience (26) *“I was a day patient and that* ***was a bit much for me***. ***So many people*** *from morning to night and you always have to talk straight away.” (1.9)* *“In the beginning, when I came here, it was so that* ***everyone was talking about their illness and I didn’t want to hear anything about illnesses.*** *It was like it for the first two weeks: “Illness, illness, illness!” (1.4)* *“I was quite content in some cases that I had some* ***distance to my fellow patients*** *. That I was able to hear and see something else, too.” (1.16)*
I.2.4. Stress experience (24) *“But also the* ***feeling of being exposed to some patients*** *whilst staying as an inpatient.” (2.1)* *“Yes, it was* ***very exhausting never really being alone*** *, never really being able to be just for myself. Then also the change to being an inpatient here; that was difficult.” (2.8)* *“Negative was that sometimes you* ***could not cope with their problems*** *. This sometimes was very stressful and I often took stories very much to heart. I then, sometimes, spent too much time on some people, and later thought, “Oh God, you shouldn’t have done that, that was too much again!* ***You’ve exceeded your own limits again.*** *” (2.19)*

3. Social contacts outside of therapy (120)
D.3.1. Support and improvement of social contacts/Positive interaction of social contacts (31) *“Simply to spend the evening at home with friends or maintaining social contacts. One’s* ***not totally removed and has to fit in again in the end.*** *That really helped me.” (1.11)* *“Actually good. I also noticed* ***that my relationship improved again*** *when I come home in the evening. That this had a positive effect. My partner also said that in the couple therapy session that I am now more relaxed and balanced. It really had a positive effect.” (1.18)* *“I thought it would actually be better to shield yourself completely, but then I realized through several therapy sessions that it was quite good also to talk to my family about such problems. This* ***also improved things a lot*** *.” (1.2)* I.3.1. Support and improvement of social contacts/Positive interaction of social contacts (42) *“So contact was* ***actually pretty good*** *. I’ve* ***done a lot with friends*** *during the stay and I was even visited by my mother and my little brother once. Yes, that was positive.” (2.4)* *“We also had a family therapy session. This […] and everything, that really helped me a lot, looking back. I* ***get along with my parents much better*** *. We can now argue and I feel much better after, than before my stay, I’d say.” (2.19)* *“With my friends far too little… but I’m just a very* ***giving person*** *. I don’t want to alienate myself. It makes you think, “Oh, after all this time! Hopefully, they haven’t forgotten me.” (2.9)*
D.3.2. Social withdrawal (18) ***“Now and again it was too much*** *. If I had, for example, talked about something that had really pulled me down, I just wanted to come home and fall into my bed… but* ***I could generally distance myself quite well from things. F*** *or example, I just didn’t answer the phone or something or I said, “I ‘m not well today, I’ll call you tomorrow, or so.” (1.7)* *“There was almost nothing, because I came home and was so tired that I often went to sleep at six or seven o’ clock. Especially, in regards to my boyfriend, I then started feeling really guilty, because then we really* ***had no more time together*** *.” (1.14)* *“With my husband, there were always problems and that* ***really got me down every time*** *. That’s why I avoided contact with him.” (1.3)* *“It actually* ***really hindered therapy*** *. It threw me back severely. It didn’t help me progress, let’s put it that way. Everything moved very slowly because of it.” (1.3)*
I.3.2. Social withdrawal (29) *“* ***I had no contact*** *. Apart from the fact that people knew that I was in the clinic, it’s like this: you get in and it’s like being on another planet. All at once, one is completely free of everything. You don’t have to worry about shopping or other things. It’s like being “beamed away.” And you’re* ***not even interested in the outside world*** *. That’s how it seemed to me, anyway.” (2.13)* *“On the weekends, when I was at home, I didn’t want to see anyone…. There is only a limited amount of time and people start hogging you. I want to have the weekend to* ***be able to sort things in my head*** *and not have to talk about it.” (2.15)* *“Because it was really important* ***for me to get the necessary distance from home*** *.” (2.6)*

4. Treatment discharge and going back to everyday life (503)
D.4.1. Leaving the unit in the evening (9) *“That’s what I wrote on the questionnaire on Friday,* ***that I find the transition from here to home really hard*** *.” (1.4)* *“… I did see it that way and also missed it somehow. As I said, it was not important to be here over night, so that I had slept here. But simply this: The others were still together, they sat together in the kitchen, or just went into town together or watched TV together in the evenings,* etc*. And in the morning they then talked about it! Well, for myself I found it was sometimes like being* ***excluded and demarked from the group*** *.” (1.13)*
I.4.1. Visiting home on the weekends (9) *“… in the early days* ***of therapy I was glad when weekends were over and I could go back again*** *. I was counting the minutes on Sunday morning until I could go back again. I just didn’t feel comfortable here.” (2.16)* *“I* ***never had the desire to go home*** *. On Saturday mornings I always felt sick. I couldn’t eat breakfast because I knew I had to go home. I always delayed going for a further 1* *h and would really have rather just stayed. And Sunday evening* ***I was always happy when I stood in front of the door*** *. I told my friends. “I’m going home again,” It just slipped out. It was home for me. My friends called and asked me, “Where are you?” and I said. “I’m on my way home” and that’s when it struck me first and I thought, “Oh my God!” (2.16)* *“If I was at home on Saturday and Sunday, I was happy again on Sunday evening when I saw my people. But then they eventually left and you* ***couldn’t go to see your mates in the clinic any longer. My friends, acquaintances, the “protective” feel around the whole thing.*** *” (2.14)*
D.4.2. Discharge from treatment (29) *“So, I’m a total opponent of the day clinic, but at the moment I’m thinking it might have been the better option for me (patient laughs).* ***It’s easier to cut that umbilical cord*** *. I mean, on the one hand I haven’t felt as attached to the other patients as I did last time because you just don’t spend as much time together.” (1.11)* *“* ***For me, it was very difficult to leave the hospital on the whole*** *. Also, because the other patients and the thought of how it all is being back.” (1.2)* *“And also,* ***this sense of security you have being in the clinic stops at four o’clock*** *…. It’s just not the same protected feeling, which one usually has for eight or ten weeks. That has made things easier. Simply keeping up your social contacts outside.” (1.11)*
I.4.2. Discharge from treatment (27) *“So I just felt that* ***the reference to normal, everyday life was a bit too small*** *. If I had still been going to school, I would have thought it would have been great if you could have something like a day’s trial. Have a day in school or a day in work. And that then discuss everything with the therapist.” (2.4)* *“This* ***feeling of being sheltered was gone*** *… it really is the case that you’re exposed to all sorts of things:* ***the thoughtfulness is no longer there*** *!* ***Everyone’s understanding is gone*** *and the worries are different, too! It’s no longer about “feeling good” or whatever, but it’s all about: “making it” and other problems. That’s a shock at first and exhausting. A change that one has to come to terms with first. This is quite a big difference.” (2.10)* *“For me, not at all. Because I thought, “When I’m out, I have to live my life.”* ***Yes, I want to live my life*** *. It scared me that whilst I was there, people were discharged and then came back every day.* ***They just could not let go*** *. Then I thought, “No, you cannot do that.* ***This is not life*** *. “We had one who came back every night. Every evening! He crept in every evening.” (2.16)*
D.4.3. Going back to everyday life (208) *“Well, the* ***routine is missing*** *, simply, having to get up in the morning and to go there. Now, it doesn’t matter. I just start the day without having a plan.” (1.17)* *“But now I realize, I haven’t used everything I’ve learned from the first week or therapy, but I realize that the potential is definitely there. And that* ***it’s not a problem to do that for me*** *.” (1.8)* *“I could do my household chores; redecorate the apartment, decorating and cooking food again…* ***all the fun in things has just come back again*** *. The enjoyment is just there again!” (1.7)* *“Before I was in the clinic, my therapist had already told me that I should get in contact with her again straight after discharge. So that we could continue my outpatient treatment again.* ***So, I knew that I had at least one contact*** *. And I also get on quite well with my GP. He really supports me.” (1.13)*
I.4.3. Going back to everyday life (221) *“It was just as it had been before: I was at my computer a lot and in bed.* ***I fell right back into my old behaviors*** *.” (2.2)* *“Actually quite positive. During my period of depression I was totally unmotivated and was doing nothing really and now you can really notice it, I’m more active, looking for an apartment, for a job and so on. So,* ***quite positive actually*** *. I’m even making plans for the next day again. That’s positive.” (2.9)* *“So, perhaps instructions to the effect: “* ***Communicate your needs*** *” This is such a nice topic. I’m certainly doing more of that now. But I’m* ***also not so much…*** ***well, this feeling of being disappointed***- *I don’t have it as much anymore. Well, a certain indifference and a certain positive distance to other people is now there.” (2.13)* *“Because I was always out and about, not really. That was perhaps an advantage.* ***I always went out and didn’t hide in my shell*** *. I went to see my friends and family as often as I could. Maybe that’s why I didn’t find it so hard now.” (2.9)*

**Table 3:** Qualitative analysis of patient statements: citations related to category 5

5. Day-clinic specific aspects. Experience of the daily commute and evenings at home (144)
D.5.1. Experience of the daily commute (40) *“It was very tiring sometimes; I* ***could hardly get here from the train station*** *… Because I was so tired and so knackered.” (1.3)* *“Well, yes, just the fact of exposing oneself to the surroundings. So, actually* ***being confronted with all your fears*** *.” (1.7)* *“I also walked; it* ***was actually really good to have a little walk*** *in the mornings. It was not particularly strenuous.” (1.8)* *“That I still* ***had to concentrate afterwards*** *. When you were sat in the car ready to drive home.” (1.17)*
D.5.2. Daily contact with the home environment (104) *“* ***Being confronted with your problems after being back in the evening,*** *with which I just couldn’t deal* – *still can’t deal with. I would have liked more time off.” (1.3)* *“* ***Other activities were, however, quite limited*** *, as I was exhausted when I came home. So if at all, then on the weekend.” (1.16)* *“I* ***didn’t see it as a double burden*** *. Because when I was working, it was also like this. I also had to do the housework. No, that was not a problem.” (1.18)* *“They are having a good time and* ***I’m alone*** *. I really had trouble with dealing with that.” (1.11)* *“One isn’t just totally torn out of everything and has to tune back in afterwards.* ***That really helped me*** *. The people, I had, still kept contact with me. From my normal surroundings. You weren’t totally out of it.” (1.11)* *“Keeping up friendships, the routine, that you have to think about how you want to spend the evenings.* ***That you’re not quite as pampered*** *, but at least have to worry about some of the meals and your household. It just means the* ***demands are*** ***different*** *. In the clinic, they really do everything for you. One only has to think and go to therapy.” (1.11)*

#### Therapeutic aspects (266)

##### D.1.1. Adjustment to the therapeutic setting (29)

Adjustment to the therapeutic setting was challenging for day clinic patients, who described experiencing difficulties in integrating themselves into the patient community as well as in beginning with the therapeutic process. In retrospect, day clinic patients felt that they had to invest a high degree of energy and initiative to integrate themselves into the pre-existing patient community. Especially patients, who had already been in hospital treatment for several weeks, were perceived as very reserved towards the new arrivals. Day clinic patients attributed early difficulties in socializing to individual personality factors and initial inhibitions towards the group. Furthermore, inhibitions in engaging in therapy and self-disclosure during group therapy were described. Particularly non-verbal treatment sessions, such as music and art therapy, were often initially perceived as disconcerting and potentially shame-inducing.

##### I.1.1. Adjustment to the therapeutic setting (20)

Overall, inpatients experienced fewer difficulties in adjusting to the therapeutic setting, reporting easy integration into the patient community and less inhibitions towards therapy engagement and self-disclosure during group therapy. Facilitated by a welcoming atmosphere and active efforts towards their integration by the unit’s existing patient community, most inpatients adjusted easily to the clinic setting. Especially periods between and after scheduled therapy sessions were seen as valuable opportunities for socialization and beneficial for their group integration. Compared to day-clinic patients, inpatients felt that their setting allowed them to experience therapy more intensely. However, some inpatients also described general difficulties in adjusting to the new environment, engaging in therapy and in opening up in front of other patients. Some also reported non-verbal treatment sessions, such as concentrated movement and art therapy, to be initially disconcerting and potentially shame-inducing.

##### D.1.2. Experience of therapy (61)

Most day clinic patients experienced treatment as beneficial, especially if they felt to have gained a better understanding of the relationship between their biography and current behaviors and emotions. Above all, patients described the body-oriented and the couple therapy sessions as well as the ‘safe haven’ experience in the hospital, giving them a stabilizing, regular daily structure, as highly beneficial. However, day clinic patients also experienced treatment as stressful listing the confrontation with intensely negative and painful emotions in the therapeutic process, as well as difficulties in learning new means of articulating concerns and needs, as reasons. Most patients felt supported by the units’ therapeutic team and described the majority of therapeutic relationships as warm and emotionally close. However, more reserved relationships and difficulties in building up trust were pointed out in some cases.

##### I.1.2. Experience of therapy (98)

Inpatients experienced therapy as predominantly beneficial and reported an improvement of depressive symptoms. The possibility of being able to spend all day in the unit and the regular daily routine was seen as particularly positive and was reported to facilitate personal activity. However, some inpatients also described difficulties in adhering to the treatment plan, which was experienced as intense and demanding with little time for relaxation. In addition, resurfacing emotions during therapy, as well as self-reflection and intended behavioral changes, were perceived as stressful. Although some patients described difficulties in building a warm and trusting relationship with therapists and nurses, relationships with the therapeutic team were predominantly perceived as benevolent and emotionally close.

##### D.1.3. Involvement of current domestic conflicts in therapy/therapy focus on social and domestic reality (21)

Day clinic patients perceived the continuous contact with everyday life routines and hassles as very autonomy-supportive and beneficial for their re-integration into everyday life after hospital discharge. In particular, the possibility to directly address interpersonal conflicts during evenings at home on the following day was seen as a significant advantage of the setting. Moreover, patients reported that they were able to directly test new skills and behaviours at home and discuss encountered difficulties during therapy the next day.

##### I.1.3. The clinic–a safe haven (37)

Inpatients described their experience of the hospital as a ‘safe haven’, shielding them from their home environment and daily hassles during the entire day, as extremely relieving and beneficial. Patients perceived the setting to allow them to focus on their own difficulties and needs more intensely and felt they were able to more fully engage themselves in the therapeutic process in consequences.

#### Patient group experience (322)

##### D.2.1. Learning through interaction (19)

Day clinic patients reported to have gained more confidence in dealing with people. In addition, they felt that the positive feedback and appreciation by fellow patients had increased their self-confidence. Moreover, patients experienced the group as a positive space in which they were able to test different or new interpersonal behaviors, such as putting down boundaries by “saying no” or self-disclosing and were thus able to overcome social anxiety or inhibitions.

##### I.2.1. Learning through interaction (19)

Inpatients felt to have improved their self-esteem through interaction with other patients and positive feedback from the group. A sense of interpersonal trust was also perceived to have been regained through patient group experiences. In addition, new skills or different behaviors, such as socializing and conversing without fear of shame or rejection, could be tested in contact with other patients.

##### D.2.2. Sharing experiences (29)

Day clinic patients saw sharing experiences and realizing that other patients faced similar difficulties in their everyday life as beneficial. Compared to their social environment, they often felt to be better understood and taken more seriously by the patient group.

##### I.2.2. Sharing experiences (34)

Shielding them from feelings of sadness and loneliness, inpatients experienced particular relief through the possibility of intense contact with the other patients In addition, they also benefited from the mutual exchange of experiences and feedback within the patient group.

##### D.2.3. Group cohesion and sense of belonging (78)

Stressing the importance of a friendly group atmosphere for positive treatment outcome, day clinic patients reported a predominantly strong sense of belonging to the units’ social environment. In regard to the inpatient community, some day clinic patients did not experience themselves as full members of the patient group, reporting feelings of greater distance and, in part, exclusion. These patients described difficulties in group integration and felt that the day clinic setting, in which patients are obliged to leave the unit by four o’clock in the afternoon, limited their possibilities of forming deeper relationships with other patients.

##### I.2.3. Group coherence and sense of belonging (93)

Inpatients in particularly reported a strong sense of emotional closeness and group coherence as well as a high degree of in-group identification. Many stated that undergoing therapy in the inpatient setting gave them a ‘special status’ and enjoyed being able to spend the evening with the other inpatients instead of having to return to feelings of loneliness at home. Inpatients emphasized their feeling of security within the inpatient group, while describing their relationship with day clinic patients as more distant. Especially the time spent together in the evenings after the daily therapy program had finished was seen as an integral part of inpatient therapy. For the patients, it served as an important basis for the development of a profound sense of belonging offering additional possibility for intensive discussions and the development of deeper relationships.

##### D.2.4. Stress experience (26)

Feeling continually exposed to fellow patients concerns, some day clinic patients experienced interactions with the other patients as stressful. Stating that the day clinical setting offered no possibility to retreat apart from the day clinic quiet room, patients described spending evenings at home as relieving as it helped them to distance themselves from other patients’ concerns.

##### I.2.4. Stress experience (24)

Some inpatients experienced the constant exposure to their fellow patients and their moods as stressful. The frequent to constant interaction with fellow patients, sometimes resulting in conflict, was seen as a difficult and challenging part of therapy.

#### Social contacts outside of therapy (120)

##### D.3.1. Support and improvement of social contacts/positive interactions with family and friends (31)

Providing them with additional ‘external’ aid during difficult times in therapy, many day-clinic patients experienced keeping in contact with family and friends during hospital treatment as supportive. Moreover, day-clinic patients felt that their interactions and contact with their partner or family improved throughout therapy as they were able to directly employ skills learned during hospital treatment, such as improved communication skills, in the evenings at home.

##### I.3.1. Support and improvement of social contacts/positive interactions with family and friends (42)

Some inpatients reported to keep in adequate and good contact with family and friends during therapy and also felt to have improved relations with their social environment as a result of skills learned and insights gained in therapy. Particularly, patients, who reported to have good social resources, expressed their desire for more contact with their social environment.

##### D.3.2. Social withdrawal (18)

Experiencing daily therapy as strenuous, some day clinic patients reported a need for rest in the evenings and to have reduced regular contact with their social environment in consequence. In addition, some patients stated that the continued confrontation with daily hassles and interpersonal conflicts at home was burdensome and, in their perception, impeded therapeutic efficacy. In particular, patients reporting conflicts at home expressed their desire for stronger shielding from their social environment through the hospital.

##### I.3.2. Social withdrawal (29)

Particularly, inpatients with fragile social backgrounds experienced the hospital unit as a shielded, ‘ideal world’ providing them with a ‘safe haven’ far away from conflicts in their home environment. Especially the shielding from social contacts was described as beneficial during therapy as this was seen to enable inpatients to focus on their own difficulties undisturbed.

#### Treatment discharge and going back to everyday life (503)

##### D.4.1. Leaving the unit in the evening (9)

Day clinic patients frequently stated difficulties with leaving the unit after the end of daily therapy. Some day clinic patients experienced the daily separation from the patient community and unit as exclusionary and reported to envy inpatients’ continued contact.

##### I.4.1. Visiting home on the weekends (9)

Some inpatients reported difficulties during the weekends spent at home and stated their desire to stay in the unit. As main difficulties, most inpatients described to feel overwhelmed by their families’ demands and lack of empathetic understanding. In particularly patients with poor social environments at home described feelings of loneliness and sadness to be most challenging over the weekends at home.

##### D.4.2. Discharge from treatment (29)

Day clinic patients reported that the daily return to their home and the continued contact to their social environment enabled them a smooth transition from hospital therapy to everyday life. Feeling that they faced fewer difficulties in emotionally and physically parting with the unit compared to inpatients, the day clinic setting facilitated discharge form therapy in their perception. However, some day clinic patients described at discharge as challenging predominantly stating difficulties in leaving the ‘safe haven’ provided by the unit and losing the regular contact with therapeutic staff.

##### I.4.2. Discharge from treatment (27)

Discharge from the unit was experienced as abrupt by many inpatients. They perceived that the relief, which had been gained through the greater distance to stressors and conflicts at home as well as from the patient community, came to a sudden end. Furthermore, many inpatients felt overwhelmed and unable to cope with everyday hassles and social isolation at the end of therapy. Though some inpatients were able to part with little difficulty, especially patients reporting feelings of loneliness at home saw discharge as challenging. Here, having to leave the unit with the knowledge that others, still in treatment, would continue to be part of the patient community was experienced as highly burdensome.

##### D.4.3. Going back to everyday life (208)

Four weeks after discharge, many day clinic patients reported to have been able to successfully transfer their learning experiences from treatment to everyday life. In addition, an improved daily structure and an enhanced sense of well-being after therapy were described. However, some patients reported to have experienced a depressive relapse and difficulties in re-entering everyday life, such as maintaining a regular daily routine or transferring learning experience, after therapy due to continued conflict in the home environment and socio-economic constraints. In addition, some patients reported feelings of loneliness after discharge as the close relationships with the unit’s other patients had ceased with the end of treatment. Here, previous social contacts and continued outpatient psychotherapy was experienced as supportive.

##### I.4.3. Going back to everyday life (221)

Some inpatients reported to have experienced little difficulty in going back to everyday life reporting significant symptom improvement, more self-confidence, improved daily structure, and good practicability of learning experiences. However, having experienced inpatient treatment as entirely detached from their life outside, the majority of inpatients felt that the transition from hospital to everyday life was difficult. Sometimes depressive symptoms, such as listlessness and fatigue, had reoccurred and difficulties in introducing and maintaining a daily structure, transferring insights and learned skills in their home environment were described. Helping them to prepare for everyday life and to avoid loneliness after treatment, patients experienced an active confrontation with the limited duration of therapy in advance as well as prearranging meetings with friends and family as beneficial.

#### Day clinic-specific aspects. Experience of the daily commute and evenings at home (144)

##### D.5.1. Experience of the daily commute (40)

Some day clinic patients described the daily commute between home and hospital as very stressful stating that the resulting exhaustion impeded their social activities. Especially patients reporting a severe lack of energy, fatigue symptoms, and concentration difficulties, or long-distance commutes, felt burdened. Despite improving as therapy progressed, some patients with comorbid social anxiety disorder or agoraphobia reported to have experienced the daily use of public transportation as highly stressful at start of therapy. Other patients valued the daily commute as a structuring element in their daily routine enabling them to prepare for or process treatment sessions.

##### D.5.2. Daily contact with the home environment (104)

Day clinic patients often experienced the daily contact with their home environment as relieving and reassuring. While many patients spent the hours in the evenings on social or leisure activities, some felt too exhausted to pursue activities after treatment. However, feeling distracted from therapy by their regular tasks and hassles at home, in particularly patients with conflicts at home experienced evenings at home as stressful. However, as family members often took on the majority of household chores during patients’ treatment, only few described the daily confrontation with their life outside the clinic as burdensome. Especially patients living alone experienced difficulties in having to return to their empty homes and feelings of loneliness in the evenings. Some of these patients reported that the lack of social contacts at night resulted in a relapse to ‘old’ behavioral patterns (e.g. staying up too late).

## Discussion

This study examined day clinic- and inpatients’ views on their psychotherapeutic treatment in an integrated hospital setting, combining day clinic and inpatient treatment on one single unit. First of all, it is important to note that there were no outcome differences between the two examined groups regarding quantitative measures for depressive symptoms and improvement of interpersonal problems as previously published by Dinger and colleagues [24, 25]. However, the current qualitative study revealed distinct differences within the RCT in regard to day clinic- and in-patients’ perceptions of the start of therapy, integration in the patient community and skill transfer at the end of treatment. Although perceived as very demanding, day clinic patients appreciated the possibility to directly address interpersonal conflicts during evenings at home on the following day. In terms of offering time to prepare for and process treatment sessions, the daily commute was experienced as beneficial and the day clinic setting was perceived to facilitate a smooth transition back to everyday life following discharge. Despite positive experiences, day clinic patients were often ambivalent towards their treatment setting as they felt burdened by the demanding requirements of a day clinic treatment approach. Inpatients felt very much relieved by their treatment setting, and well integrated into the patient group. Start of therapy, particularly self-disclosure during group therapy, was perceived as easy. Furthermore, inpatients were not only convinced of their treatment setting’s effectiveness but also appeared to be sceptical towards day clinic treatment. However, despite inpatients’ positive views of their own therapy setting, they struggled severely with the transfer to everyday life after discharge. In the following section, we will discuss the most prominent differential aspects of the two treatment settings in detail.

All day clinic patients reported difficulties with initial integration into the existing patient group. In their experience, they had to be very proactive in order to feel as part of the patient group. Inpatients, in contrast, reported facilitated patient group integration due to the extensive amount of time spent with fellow inpatients during the evenings. In their experience, more time spent in the hospital led to greater trust and earlier self-disclosure both during group therapies and during informal discussions outside of therapy sessions. These findings contradict those of Eichler et al. [22], who reported that patients experienced inpatient and day clinic treatment as equally positive and effective. However, day clinic and inpatients were treated on separated units in their study and were not able to observe the other group directly. Furthermore, the standard psychiatric care in Eichler’s study was less focused on psychotherapy and group processes.

The patient community was perceived as very supportive during further course of treatment across settings. Learning from other patients’ experiences, having close social relationships again, and benefitting from other patients’ feedback were listed as highly relevant aspect for the success of their treatment. Within their setting, inpatients developed a high sense of group cohesion due to “around-the-clock” therapy, but were reluctant in developing an intimate atmosphere with day clinic patients. Day clinic patients, in contrast, felt excluded and somewhat disadvantaged. The fact that day clinic patients were obliged to leave the hospital every day by 4 p.m. reinforced these exclusionary feelings. In regard to the findings on group cohesion, the integrated therapy setting, in which day clinic- and in-patients are treated together, is of specific importance. In the current study, the integrated setting was a major advantage for the scientific comparison of the two treatments as variables, such as therapy components and therapists, could be kept constant between conditions (high internal validity). Although not in the focus of the current investigation, the integrated setting is also ideal for the investigation of changes of treatment intensity during therapy (“step up” and “step down”). However, in an integrated setting, the possibility of direct comparison with inpatients, who seemingly receive “more treatment,” appears to be a specific challenge for day-clinic patients. Based on previous research on the importance of group cohesion within a therapeutic community [23, 36, 37], our findings call for special attention to the day clinic patients’ integration in the case of a mixed-setting therapeutic patient community. Therapeutic interventions in cross-setting subgroups might support and foster intergroup relations.

Day clinic patients perceived the daily commute as a further burden in addition to the demanding psychotherapy. In some cases, this extra strain impeded social and leisure activities or other positive resources at home due to exhaustion. It is likely that the daily commute is particularly challenging for depressive patients, as loss of energy represents one of the major diagnostic criteria [38]. In their non-randomized, observational INSTAP study, Zeeck et al. [9] found a significant negative relationship between loss of energy and clinical outcome for day clinic patients. Therefore, the question whether or not a significant loss of energy is a specific hindrance for day clinic therapy requires further attention. While Wietersheim et al. [13] reported that patients with anxiety disorders feel overburdened by the daily commute, some depressive day clinic patients with comorbid anxiety disorders in our study described a reduction of psychosocial fears when using public transport over the course of therapy. However, the careful evaluation of individual patient’s abilities and setting demands remains imperative.

Regarding psychotherapeutic treatment itself, both patient groups reported that therapy was demanding but at the same time beneficial for reflecting feelings and behavior. Patients predominantly described positive relationships with the professional psychotherapeutic team, only some day clinic patients reported to have experienced a more distant relationship. Inpatients reported that being sheltered from their social environment represented a key factor to and basis of their therapy success. However, the underlying explanatory models for this experience differed within the inpatient group. Some inpatients highlighted their desire to gain distance from severe conflicts at home, while others emphasized their wish for a trouble-free ‘safe haven’. The latter often struggled with feeling of loneliness after discharge. In contrast, day clinic patients experienced contact with their social environment far more positively, especially when there were no major conflicts at home. In these cases, they described their partners, relatives or friends as a source of support. In cases of conflicts within their social environment, day clinic patients tended to perceive themselves as overburdened. Accordingly, the level of social conflicts should be taken into account, when evaluating the differential indication for day clinic- versus in-patient treatment settings.

Focusing on the end of therapy and skill and insight transfer to everyday life, both patient groups reported that they experienced difficulties when leaving the sheltering therapy unit. However, this challenge was perceived differently in both groups. Day clinic patients emphasized the experience of loss and sadness when having to leave the safe haven of the hospital and their attachment figures during therapy, while inpatients reported greater concerns in regard to having to abruptly face their everyday life again. The sheltered setting of inpatient treatment appears to promote the desire to be taken care of (pronounced dependency) which is also reflected in the frequent wish to remain in the trouble-free unit during weekends. On the other hand, the intensive support during the limited treatment time may promote the internalization of positive relationship experiences, especially for patients with increased self-reliance. In successful inpatient therapies, these positive relationship experiences appear to strengthen patients’ self-view and allow a more confident re-entry into their everyday life. Day clinic patients, by contrast, ‘practice’ and experience parting from the patient group every day during treatment. Wietersheim et al. [13] assumes that day clinic treatment counteracts tendencies for pronounced dependency and regression, which might ease re-entry into everyday life. Furthermore, day clinic patients quickly learn that therapists are not able to provide all-embracing care, having to resume responsibility for their own life at an early treatment stage in consequence [39]. Some day clinic patients, however, were envious of other patients who were still allowed to stay in the hospital, while they were back at home. Inpatients who relapsed during the 4-week follow-up experienced the e stark change from the hospital to everyday life as triggering. Accordingly, previous authors have proposed a ‘step-down-approach’ with inpatient care being followed by day clinic treatment to facilitate a smoother transition for inpatients [9]. When focusing on the transfer of therapeutic insights and skills to everyday life, day clinic patients highlighted that transfer was eased by the possibility of discussing interpersonal conflicts at home during therapy the next day. This supports previous findings by Zeeck et al. [14]. Von Wietersheim et al. [13] showing that the recurrent “escape” from the home environment to the sheltered hospital was perceived as a major benefit of the day clinic setting. In a previous qualitative study using semi-structured interviews, the successful transfer of insights and skills from therapy to everyday life was shown to be a crucial factors for day clinic patients [8]. Furthermore, both groups experienced a prescheduled appointment with the subsequent outpatient therapist as a further beneficial and security-enhancing factor after discharge.

### Limitations

Some limitations of our study should be noted. Firstly, potentially biasing our analysis, our study is limited by the small number of participants due to its qualitative approach. However, the risk of bias is reduced by the fact that all patients, who completed the randomized-controlled trial by Dinger and colleagues [24, 25], participated in the interviews. Alsoto participate , since only 44 out of 140 eligible patient participants decided in the randomized clinical trial, it has to be noted that the current sample represents a selected group of participants. This limits the generalizability of our findings. Secondly, our results are limited to integrated settings, in which day clinic- and in-patients are treated together. However, suggesting that some of the observed effects may be specific to integrated settings, previous evaluations of separated day clinic- and in-patient treatment units have shown mixed results reporting either no differences between settings, or a patient preference for day clinic treatment [22, 40–42] Lastly, although the qualitative content analysis was performed according to principles of inductive category development and was verified by a second researcher, the examination can be considered to be less generalizable than quantitative approaches due to the subjective nature of qualitative studies. However, with the aim of drawing a more complete picture of this multilayered topic potentially identifying new research aspects, this methodological approach was specifically chosen to elucidate day clinic- and in-patients’ perceptions of treatment settings.

## Conclusions

In line with previous research, this study provides further indication that depressed patients perceive the day clinic treatment’s main advantage in the possibility to address current conflicts, while simultaneously being able to test proposed solutions in the home environment. However, in our integrated setting, day clinic patients reported difficulties in patient group integration and development of group cohesion. Inpatients saw their main advantage in the sheltered setting, distance from their social environment and maximum patient group support. However, results also show that inpatients with pronounced dependency and low interest in keeping in contact with their social environment experienced more difficulties going back to everyday life and increased depressive symptoms after discharge. For depressed patients, the clinical indication for day clinic therapy requires a careful consideration of potential difficulties. Our study identified overburden by the daily commute, the continuous stay in a potentially dysfunctional environment and interpersonal problems as risk factors for a successful patient integration. Hence, integrated models providing the possibility of a flexible step-up as well as step-down approach may be especially beneficial for personalized treatment planning in intensive settings [13]. At the same time, further research on day clinic- and in-patient treatment could benefit from our study’s results with particular focus on the transition from hospital treatment to everyday life.

## Abbreviations

RCTs: randomized controlled trials
MDE: major depressive episode
TA: thematic analysis
DSM: diagnostic and statistical manual for mental disorders
